# Synthesis, characterization, DFT calculations, and crystal structure of [Ru_2_(O_2_CCF_3_)_2_(CO)_4_*L*_2_]: tri­fluoro­acetate-bridged dimeric ruthenium(I) sawhorse complexes bearing phosphine ligands

**DOI:** 10.1107/S2056989026006316

**Published:** 2026-06-23

**Authors:** Dustin C. Brown, Isaac J. Stern, Richard J. Staples, Alexander R. Keim, Yvonne Nguyen, Thomas J. Malosh

**Affiliations:** a University of Pittsburgh Johnstown, Department of Chemistry, 450 Schoolhouse Rd, Johnstown, PA 15904, USA; b Michigan State University, Department of Chemistry and Chemical Biology, East Lansing, MI 48824, USA; University of Missouri-Columbia, USA

**Keywords:** crystal structure, dimeric ruthenium, Ru—Ru bond, sawhorse complexes, carboxyl­ate bridges, perfluorinated, DFT optimizations

## Abstract

The title compound bis­(μ-tri­fluoro­acetato-κ^2^*O*:*O*′)bis­{dicarbon­yl[tris­(penta­fluoro­phen­yl)phosphine-κ*P*}diruthenium(I)(*Ru*—*Ru*), [Ru_2_(O_2_CCF_3_)_2_(CO)_4_{P(C_6_F_5_)_3_}_2_] (**1**), has been synthesized by two protocols and its structure has been determined. Compound **1** belongs to a category of dimeric ruthenium complexes that have been shown to catalyze a number of organic, small mol­ecule transformations. In particular, the observed alkene isomerization activity of [Ru_2_(O_2_CCH_3_)_2_(CO)_4_{P(C_6_F_5_)_3_}_2_] (**2**) led to the synthesis of **1**, which differs only by its tri­fluoro­acetate bridges. Replacing the terminal, perfluorinated phosphine ligands of **1** and **2** with tri­phenyl­phosphine results in the previously reported complexes [Ru_2_(O_2_CCF_3_)_2_(CO)_4_(PPh_3_)_2_] (**3**) and [Ru_2_(O_2_CCH_3_)_2_(CO)_4_(PPh_3_)_2_] (**4**). Summary crystal data, spectroscopic characteristics, and DFT calculated parameters are presented for **1**–**4**.

## Abstract 2

The compound [Ru_2_(O_2_CCF_3_)_2_{P(C_6_F_5_)_3_}_2_(CO)_4_] (**1**), has been synthesized by two protocols and its structure has been determined. Various structural, spectroscopic, and DFT calculated parameters of (**1**), and a group of related compounds **2**–**4**: [Ru_2_(O_2_CCH_3_)_2_(CO)_4_{P(C_6_F_5_)_3_}_2_] (**2**), [Ru_2_(O_2_CCF_3_)_2_(CO)_4_(PPh_3_)_2_] (**3**), and [Ru_2_(O_2_CCH_3_)_2_(CO)_4_(PPh_3_)_2_] (**4**), are presented.

## 1. Chemical context

Phosphine-supported diruthenium tetra­carbonyl carboxyl­ates are known to exhibit catalytic activity toward several small mol­ecular transformations. The catalyzed reactions include alkene isomerization (Rohrabaugh Jr *et al.*, 2016 ▸; Salvini *et al.*, 1994 ▸), the hydrogenation of alkenes under scCO_2_ conditions (Johnpeter *et al.*, 2013 ▸), the semi-hydrogenation of diaryl alkynes (Li & Hua, 2011 ▸), the conversion of acetic acid to ethyl acetate and methanol (Salvini *et al.*, 2005 ▸), the hydrogenation of alkenes and ketones (Matteoli *et al.*, 1995 ▸), the hydro­formyl­ation of alkenes (Salvini *et al.*, 1994 ▸; Kalck *et al.*, 1991 ▸), the benzyl­ation of phenol (Jaouhari, 1994 ▸), and the conversion of dimethyl oxalate to methyl glycolate and ethyl­ene glycol (Matteoli *et al.*, 1991 ▸). Within the context of alkene isomerization, two studies report that a correlation was observed between the P—Ru—Ru—P torsion angle and catalytic activity (Matteoli *et al.*, 1995 ▸; Salvini *et al.*, 2000 ▸). The second study also postulated a mechanism that initially involves the substitution of 1-hexene for one terminal phosphine ligand to form an η^2^-1-hexene inter­mediate (Salvini *et al.*, 2000 ▸). Both studies implicate the steric effects of the terminal phosphine ligands. A third study employed a unique set of terminal phosphine ligands, and found no correlation between the P—Ru—Ru—P torsion angle and catalytic activity. The results indicate that the σ-donating ability of the phosphine may influence alkene isomerization activity (Rohrabaugh Jr *et al.*, 2016 ▸). With the fluorination of the acetate bridges, complexes involved in the third study are provided with novel electronic environments.
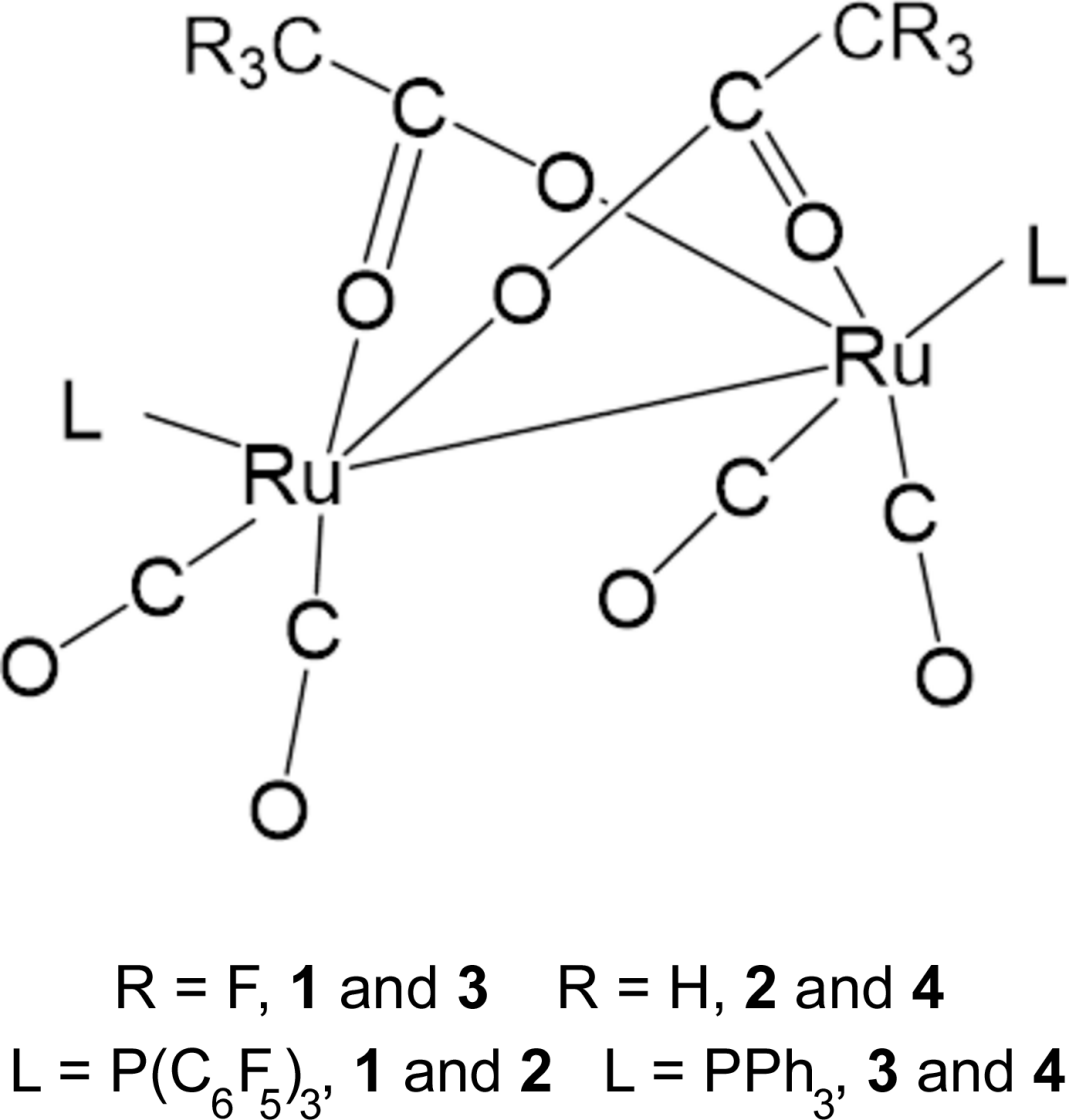


## 2. Structural commentary

**Figure 1:**
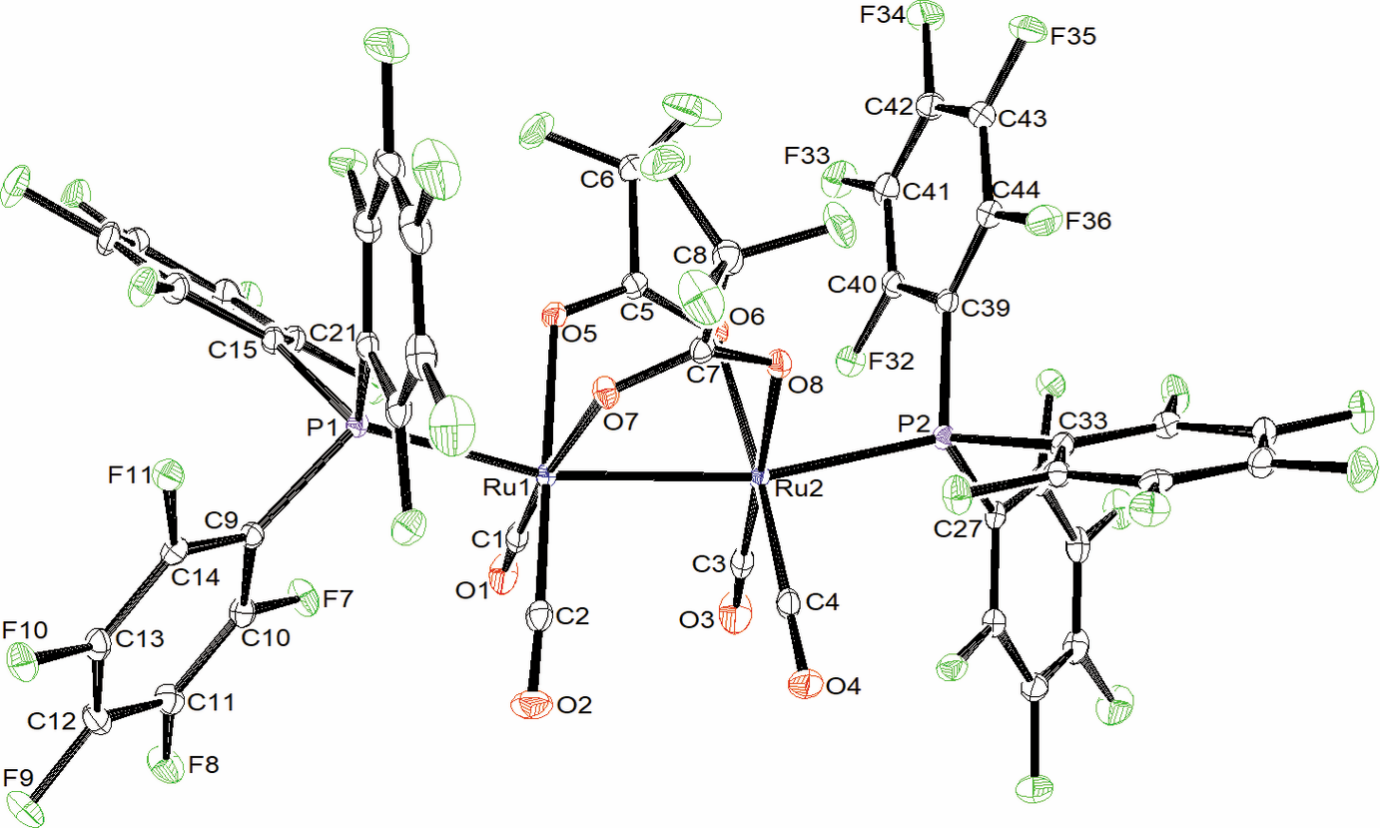
The mol­ecular structure of (**1**) with labeling and displacement ellipsoids drawn at the 40% probability level.>

The mol­ecular structure of **1** is presented in Fig. 1 ▸. Relevant parameters are presented in Table 1 ▸. Each ruthenium atom is located at the center of an irregular octa­hedron formed by coordination to five ligands, with the sixth coordination site occupied by the ruthenium-ruthenium bond. The 2-electron donating axial phosphine ligands are located *trans* to the Ru—Ru bond, with each ruthenium-phospho­rus bond being roughly, but not exactly, collinear to the Ru—Ru bond. The P—Ru—Ru—P torsion angle is 2.13°. The equatorial plane around each ruthenium center consists of a pair of μ-acetate ligands along with a pair of carbon monoxide ligands, where both pairs of ligands are mutually *cis*. The idealized structure exhibits *C_2v_* point-group symmetry, and also illustrates the sawhorse configuration of these complexes. The idealized structure is achieved by **1** in the solid state, Fig. 1 ▸. The idealized structure is also realized by **2**–**4** in the solid state (Rohrabaugh Jr *et al.*, 2016 ▸; Kom-Bei *et al.*, 1993 ▸).

The phosphine cone angles, as exhibited by the solid-state structures, have been calculated following the method advanced by Müller & Mingos (1995 ▸). As listed in Table 1 ▸, the calculated phosphine cone angles are comparable for both pairs of complexes. The calculated cone angles for the two P(C_6_F_5_)_3_ complexes differ by 1°, at 168 and 167°, respectively. The calculated cone angles for both PPh_3_ adducts also show a 1° spread, at 155 and 154°, respectively. However, there are differences between the calculated phosphine cone angles and the Tolman cone angles (Tolman, 1977 ▸). The P(C_6_F_5_)_3_ cone angles are approximately 15° smaller than Tolman, while the PPh_3_ cone angles are *circa* 10° larger than Tolman.

The ideal *C*_2*v*_ sawhorse configuration implies a P—Ru—Ru—P torsion angle of 0°, eclipsed carbon monoxide legs, and strictly planar carboxyl­ate bridges. From the various torsion angles presented in Table 1 ▸, it is apparent that solid-state structures usually deviate from the ideal. For the published compounds referenced in the *Database survey*, reported P—Ru—Ru—P torsion angles range from 2.5 (7) to 82.89 (18)° for the P^*i*^Pr_3_ and P(*o*-tol­yl)_3_ complexes, respectively (Matteoli *et al.*, 1995 ▸; Rohrabaugh Jr *et al.*, 2016 ▸). From current and previously reported data, there is no correlation between the phosphine cone angle and the torsion angle along the backbone of these diruthenium sawhorse complexes (Matteoli *et al.*, 1995 ▸; Rohrabaugh Jr *et al.*, 2016 ▸).

However, one study, above, states that steric effects involving the phosphines and carboxyl­ate groups are the likely cause of the P—Ru—Ru—P torsion angles (Matteoli *et al.*, 1995 ▸). The second study, above, reports a number of non-bonding inter­actions, both intra- and inter-mol­ecular. Non-bonding, intra­molecular inter­actions were observed between terminal phosphine ligands and bridging acetate groups. In addition, these solid-state structures exhibited a variety of aromatic inter­actions (Rohrabaugh Jr *et al.*, 2016 ▸).

## 3. Spectroscopic characterization

Compounds **1**–**4** are yellow, air stable, crystalline powders, which have been characterized by infrared, proton NMR (**2**,**4**), fluorine-19 NMR (**1**,**3**), phospho­rus-31 NMR, and elemental analysis. The IR bands, in the 2200–1800 cm^−1^ region exhibited by **1**–**4** (pairwise) are listed in Table 2 ▸, along with relevant phospho­rus-31 NMR results. The infrared spectra of **1**–**4** exhibit the expected pattern of three (strong–medium–strong) bands attributed to carbonyl stretching, and zero to three additional weak bands/shoulders in the 2200–1800 cm^−1^ region. These carbon monoxide stretching frequencies display an expected trend that inversely correlates to the σ-donating abilities of the terminal phosphine ligands, *L*. The observed trend is *v*(CO):P(C_6_F_5_)_3_ > *v*(CO):PPh_3_. Also expected and observed is the effect on carbon monoxide stretching of the terminal moiety CF_3_*versus* CH_3_ on the bridging acetate ligands where all *v*(CO) of **1** are at higher frequencies than those of **2**, and all *v*(CO) of **3** are at higher frequencies than those of **4**. Every infrared spectrum features at least two bands assigned to the symmetric and asymmetric stretching of the carboxyl­ate bridges, found in the 1600–1400 cm^−1^ region.

The ^31^P NMR spectra of **1-**-**4** each consists of a single signal. Inter­estingly for **1**–**4**, the ΔP(ppm) of the complex *versus* the free ligand follows the same trend as *v*(CO). For compounds **1** and **2**, the average ^31^P NMR coordination chemical shift is 45.78 ppm, while for compounds **3** and **4**, the average ^31^P NMR coordination chemical shift is 20.54 ppm. Within these four complexes, there is an inverse relationship between the σ-donating abilities of the terminal phosphine ligands and the magnitude of the ^31^P NMR coordination chemical shift. The ^1^H NMR spectra of **2** and **4** contain one singlet assigned to the bridging acetate ligands. Characteristic signals are found in the aromatic regions of the ^1^H NMR spectra of **3** and **4**. The ^19^F NMR spectra of **1**–**3** are similar to the ^1^H NMR spectra. The ^19^F spectra of **1** and **2** both exhibit three signals corresponding to the *ortho*, *meta*, and *para* fluorine substituents, respectively. The assignment of each signal to a specific ring position is based on both integration, and previous reports (Hogben & Graham, 1969 ▸). Finally, there is an observed singlet in the ^19^F NMR spectra of both **1** and **3** assigned to the CF_3_ moiety on the bridging tri­fluoro­acetate ligands.

The infrared and NMR data support a solution configuration for **1**–**4** in which the terminal ligands and both of the bridging acetate ligands are symmetrically equivalent. Such equivalencies are achieved by the *C*_2*v*_ sawhorse configuration of [Ru_2_(μ-O_2_CC*R*_3_)_2_(CO)_4_*L*_2_] in which the pairs of eclipsed, *cis*-carbonyl groups are the legs of the sawhorse. Thus, the solution configuration of **1**–**4** is consistent with the solid-state configuration, as illustrated in Fig. 1 ▸.

## 4. Density functional theory calculations

The backbone torsion angles (P—Ru—Ru—P)° of sawhorse complexes in the solid state warrant the calculation of optimized mol­ecular geometries for **1**–**4**. Based on the literature, the Perdew, Burke, and Ernzerhof functional (PBEPBE) was employed. Selected results, shown in Table 3 ▸, were provided by *Gaussian09* using the Perdew, Burke, and Ernzerhof functional, with the Stuttgart/Dresden ECP for ruthenium, and a 6-31G(d) basis for any additional elements (Frisch *et al.*, 2009 ▸). For all models, the computations provided zero imaginary frequencies.

The geometric parameters of the gas-phase mol­ecules exhibit some correlations to solid-state observations. The Ru—Ru bond lengths are in agreement to ∼0.01 Å. Also similar are the average Ru—P—C bond angles, and therefore the calculated terminal phosphine cone angles. However, there is a lack of correlation between calculated and solid-state P—Ru—Ru—P torsion angles.

## 5. Supra­molecular features

**Figure 2:**
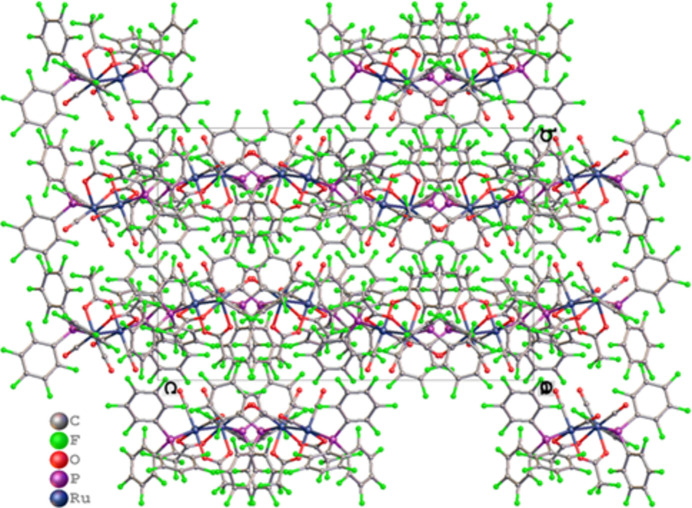
Crystal packing of (**1**) along the crystallographic *a*-axis direction.

Compound **1** is perfluorinated. The structure is devoid of hydrogen atoms. There is no π stacking. The packing is illustrated in Fig. 2 ▸.

## 6. Database survey

A number of diruthenium tetra­carbonyl compounds featuring bis­(acetato) bridges have been characterized by X-ray crystallography. Those structures that also incorporate phosphine ligands [ZUZJUB, ZUZKAI, ZUZKEM (Rohrabaugh Jr *et al.*, 2016 ▸); WAPQUB (Li & Hua, 2011 ▸); VUFMOZ (Malosh *et al.*, 2009 ▸); MUCVOW (Štěpnička & Císařová, 2009 ▸); NIMZUE (Field *et al.*, 1997 ▸); ZERROD, ZERRUJ, ZERSAQ (Matteoli *et al.*, 1995 ▸); WATFAY, WATFIG (Kom-Bei *et al.*, 1993 ▸); SEDKOB (Bright *et al.*, 1988 ▸)]. There is a single reported structure of a bis­(tri­fluoro­acetato) diruthenium compound employing a terminal phosphine ligand (Kom-Bei *et al.*, 1993 ▸). Compound **1** is the second such bis­(tri­fluoro­acetato) diruthenium compound, while being the first perfluorinated example.

## 7. Synthesis and crystallization

Preparations were performed under nitro­gen atmospheres employing dual gas/vacuum manifolds and standard Schlenk techniques. The various phosphine ligands were used as received from either Sigma-Aldrich or Strem and were manipulated in glove bags under nitro­gen atmospheres. Organic solvents meeting ACS specifications, or better, were employed and were degassed and saturated with dry nitro­gen prior to use. The compounds [Ru_3_(CO)_12_], and tri­fluoro­acetic acid were obtained from Sigma-Aldrich and were used as received. The synthesis of **2**, [Ru_2_(O_2_CCH_3_)_2_(CO)_4_{P(C_6_F_5_)_3_}_2_] is reported elsewhere (Rohrabaugh Jr *et al.*, 2016 ▸). The syntheses of **3** and **4**, the tri­phenyl­phosphine adducts, [Ru_2_(O_2_CC*X*_3_)_2_(CO)_4_(PPh_3_)_2_], where *X* = F (**3**) and *X* = H (**4**), were achieved by following previously published protocols (Bruce *et al.*, 1999 ▸; Crooks *et al.*, 1969 ▸). The recrystallization of compound **1** was performed under aerobic conditions.

Infrared spectra of starting materials and synthetic targets were recorded on a Perkin-Elmer Spectrum Two FTIR instrument. A Bruker Ascend 400 MHz FT NMR instrument was employed to obtain ^1^H, ^19^F{^1^H}, and ^31^P{^1^H} spectra of both starting materials and synthetic targets. The ^19^F and ^31^P chemical shifts are reported *versus* C_6_F_6_ and 85% H_3_PO_4_, respectively. Single crystal X-ray structural analyses were performed at the Center for Crystallographic Research at Michigan State University, Department of Chemistry, East Lansing, MI, USA. Elemental Analyses were performed at Atlantic Microlab, Inc., located in Norcross, GA, USA.

Complex **1** was prepared via two unique protocols, the first in the manner of Kalck (Kalck *et al.*, 1991 ▸). [Ru_3_(CO)_12_], 200 mg (0.313 mmol), 25 mL of sparged benzene, and tri­fluoro­acetic acid (2 mmol) were added to a three-neck 100 mL round-bottom flask (RBF) with condenser and gas inlet. The mixture was stirred and heated at 338 K for 40 h. The contents of the RBF were filtered on a glass frit. The solvent was then removed *in vacuo* to obtain a pale orange solid. The pale orange product (89% yield) and two equivalents of P(C_6_F_5_)_3_ (0.84 mmol) were added to 25 mL of sparged toluene in a three-neck 100 mL RBF with condenser and gas inlet. The mixture was stirred and heated at 343 K for 18 h. The toluene was removed *in vacuo* to yield a yellow oil. The yellow oil was dissolved in minimal chloro­form. Under ambient conditions, a layer of ethanol was allowed to slowly diffuse into the chloro­form solution. The resulting yellow crystals were collected on a fritted glass crucible, washed with chilled ethanol, and dried under vacuum. Yield: (341 mg, 0.213 mmol, 51%). IR (CHCl_3_, cm^−1^) ν(CO): 2061 (*s*), 2022 (*m*), 1997 (*s*), 1971 (*w*); ν(CO_2_): 1660 (*m*), 1642 (m). ^19^F{^1^H} NMR (CDCl_3_) δ: −75.48 (*s*, 6F, CF_3_), −126.60 (*d-br*, 12F, *o*), −144.46 (*d-br*, 6F, *p*), −155.09 (*t*, 12F, *m*). ^31^P{^1^H} NMR (CDCl_3_) δ: −28.11 (*s*). Analysis calculated for C_44_F_36_O_8_P_2_Ru_2_: C, 32.94; F, 42.63. Found: C, 32.89; F, 42.41%.

Complex **1** was also prepared in the manner of Skelton (Bruce *et al.*, 1999 ▸). [Ru_3_(CO)_12_], 100 mg (0.156 mmol), 10 mL of aceto­nitrile, tri­fluoro­acetic acid (140 mg, 1.2 mmol), and 25 mL of sparged di­chloro­methane were added to a three-neck 100 mL RBF with condenser and gas inlet. The mixture was heated at reflux for 16 h. The residue was not isolated and was redissolved in 25 mL of sparged toluene, to which was added two equivalents of P(C_6_F_5_)_3_ (0.47 mmol). The mixture was stirred and heated at 343 K for 4 h. The toluene was removed *in vacuo* to yield a yellow powder. The reaction residue was dissolved in minimal di­chloro­methane. Under ambient conditions, a layer of ethanol was allowed to slowly diffuse into the di­chloro­methane solution. The resulting yellow crystals were collected on a fritted glass filter, washed with chilled ethanol, and dried under vacuum. Yield: (235 mg, 0.146 mmol, 62%). Analysis calculated for C_44_F_36_O_8_P_2_Ru_2_: C, 32.94; F, 42.63. Found: C, 32.92; F, 42.40%.

## 8. Refinement

Crystal data, data collection, and structure refinement details are summarized in Table 4 ▸. The structure was refined by Least-Squares *SHELXL* incorporated in the *Olex2* software program (Sheldrick, 2015*b* ▸; Dolomanov, *et al.*, 2009 ▸). All non-hydrogen atoms were refined anisotropically. There are no hydrogen atoms in this mol­ecule.

## Supplementary Material

Crystal structure: contains datablock(s) I. DOI: 10.1107/S2056989026006316/ev2029sup1.cif

Structure factors: contains datablock(s) I. DOI: 10.1107/S2056989026006316/ev2029Isup2.hkl

CCDC reference: 2535396

Additional supporting information:  crystallographic information; 3D view; checkCIF report

## Figures and Tables

**Table 1:** Crystal data: selected bond lengths, torsion angles and angles (Å, °) for **1**–**4**^*a*^

Adduct	**1** CF_3_/P(C_6_F_5_)_3_	**2** CH_3_/P(C_6_F_5_)_3_	**3** CF_3_/PPh_3_	**4** CH_3_/PPh_3_
Ru—Ru	2.7276 (7)	2.6918 (2)	2.7276 (9)	2.7360 (9)
Ru—P	2.4428 (7)	2.4370 (6)	2.4461 (21)	2.4508 (10)
P—C avg.	1.838 (2)	1.840 (3)	1.824 (7)	1.824 (1)
P—Ru—Ru—P	2.13 (15)	19.11 (14)	24.07 (11)	21.14 (11)
O—Ru—Ru—O^*b*^	12.21 (5)	13.61 (7)	14.89 (16)	1.47 (8)
C—Ru—Ru—C^*c*^	11.11 (9)	17.00 (13)	20.27 (22)	2.67 (14)
Ru—P—C avg.	115.83 (6)	115.64 (9)	114.5 (3)	115.1 (3)
Cone Angle^*d*^	168	167	155	154
Cone Angle^*e*^	184	184	145	145

Notes: (*a*) **1** this work, **2** Rohrabaugh Jr *et al.* (2016 ▸), **3**–**4** Kom-Bei *et al.* (1993 ▸); (*b*) avg. OAc bridges; (*c*) avg. CO ligands; (*d*) calc. avg. Müller *et al.* (1995 ▸); (*e*) Tolman (1977 ▸).

**Table 2:** Selected spectroscopic data for **1**–**4**

	IR ν(CO)^*a*^	cm**^−^**^1^			^31^P{^1^H}^*b*^	
Adduct:	(*s*)	(*m*)	(*s*)	(*w*)	δ ligand	δ complex
CF_3_/P(C_6_F_5_)_3_	2061	2022	1997	1971	−74.26	−28.11
CH_3_/P(C_6_F_5_)_3_	2047	2008	1979	1953	−74.26	−28.85
CF_3_/PPh_3_	2037	1994	1968	1941	−5.41	15.87
CH_3_/PPh_3_	2023	1978	1949	1919	−5.41	14.39

Notes: (*a*) in CHCl_3_; (*b*) *vs* H_3_PO_4_.

**Table 3:** Optimized models: geometric parameters (Å, °) for **1**–**4**^*a*^

Complex	**1**	**2**	**3**	**4**
Ru—Ru	2.716	2.684	2.731	2.700
Ru—P	2.437	2.423	2.436	2.437
P—C avg.	1.840	1.842	1.834	1.836
P—Ru—Ru—P	7.3	29.9	27.3	43.5
O—Ru—Ru—O^*b*^	14.3	17.0	15.8	18.5
C—Ru—Ru—C^*c*^	11.6	24.1	21.5	24.9
Ru—P—C avg.	115.3	115.5	114.7	115.1
Cone Angle^*d*^	169	169	157	158
Cone Angle^*e*^	184	184	145	145

Notes: (*a*) PBE0/MWB28; (*b*) avg. OAc bridges; (*c*) avg. CO ligands; (*d*) calc. avg. Müller *et al.* (1995 ▸); (*e*) Tolman (1977 ▸).

**Table 4:** Experimental details

Crystal data	
Chemical formula	[Ru_2_(C_2_F_3_O_2_)_2_(C_18_F_15_P)_2_(CO)_4_]
*M*_r_	1604.52
Crystal system, space group	Orthorhombic, *Pbca*
Temperature (K)	100
*a*, *b*, *c* (Å)	20.2997 (1), 18.1382 (1), 27.0110 (1)
*V* (Å^3^)	9945.45 (8)
*Z*	8
Radiation type	Cu *K*α
μ (mm^−1^)	7.33
Crystal size (mm)	0.18 × 0.18 × 0.04

Data collection	
Diffractometer	XtaLAB Synergy, Dualflex, HyPix
Absorption correction	Gaussian (*CrysAlis PRO*; Rigaku OD, 2022 ▸)
*T*_min_, *T*_max_	0.586, 1.000
No. of measured, independent and observed [*I* > 2σ(*I*)] reflections	145168, 10842, 10581
*R*_int_	0.042
(sin θ/λ)_max_ (Å^−1^)	0.639

Refinement	
*R*[*F*^2^ > 2σ(*F*^2^)], *wR*(*F*^2^), *S*	0.023, 0.060, 1.07
No. of reflections	10842
No. of parameters	829
Δρ_max_, Δρ_min_ (e Å^−3^)	0.47, −0.75

Computer programs: *CrysAlis PRO* (Rigaku OD, 2022 ▸), *SHELXT2014/5* (Sheldrick, 2015*a* ▸), *SHELXL2018/3* (Sheldrick, 2015*b* ▸) and *OLEX2* (Dolomanov *et al.*, 2009 ▸.

## supplementary crystallographic information

### Bis(µ-trifluoroacetato-κ2O:O')bis{dicarbonyl[tris(pentafluorophenyl)phosphine-κP}diruthenium(I)(Ru—Ru) Crystal data

**Table:**

[Ru_2_(C_2_F_3_O_2_)_2_(C_18_F_15_P)_2_(CO)_4_]	*D*_x_ = 2.143 Mg m^−^^3^
*M**_r_* = 1604.52	Cu *K*α radiation, λ = 1.54184 Å
Orthorhombic, *P**b**c**a*	Cell parameters from 91708 reflections
*a* = 20.2997 (1) Å	θ = 3.3–79.9°
*b* = 18.1382 (1) Å	µ = 7.33 mm^−^^1^
*c* = 27.0110 (1) Å	*T* = 100 K
*V* = 9945.45 (8) Å^3^	Rhombohedral, yellow
*Z* = 8	0.18 × 0.18 × 0.04 mm
*F*(000) = 6160	

### Bis(µ-trifluoroacetato-κ2O:O')bis{dicarbonyl[tris(pentafluorophenyl)phosphine-κP}diruthenium(I)(Ru—Ru) Data collection

**Table:**

XtaLAB Synergy, Dualflex, HyPix diffractometer	10842 independent reflections
Radiation source: micro-focus sealed X-ray tube, PhotonJet (Cu) X-ray Source	10581 reflections with *I* > 2σ(*I*)
Mirror monochromator	*R*_int_ = 0.042
Detector resolution: 10.0000 pixels mm^-1^	θ_max_ = 80.3°, θ_min_ = 3.3°
ω scans	*h* = −25→25
Absorption correction: gaussian (CrysAlisPro; Rigaku OD, 2022)	*k* = −23→23
*T*_min_ = 0.586, *T*_max_ = 1.000	*l* = −34→29
145168 measured reflections	

### Bis(µ-trifluoroacetato-κ2O:O')bis{dicarbonyl[tris(pentafluorophenyl)phosphine-κP}diruthenium(I)(Ru—Ru) Refinement

**Table:**

Refinement on *F*^2^	0 restraints
Least-squares matrix: full	Primary atom site location: dual
*R*[*F*^2^ > 2σ(*F*^2^)] = 0.023	*w* = 1/[σ^2^(*F*_o_^2^) + (0.0307*P*)^2^ + 10.1377*P*] where *P* = (*F*_o_^2^ + 2*F*_c_^2^)/3
*wR*(*F*^2^) = 0.060	(Δ/σ)_max_ = 0.005
*S* = 1.07	Δρ_max_ = 0.47 e Å^−^^3^
10842 reflections	Δρ_min_ = −0.75 e Å^−^^3^
829 parameters	

### Bis(µ-trifluoroacetato-κ2O:O')bis{dicarbonyl[tris(pentafluorophenyl)phosphine-κP}diruthenium(I)(Ru—Ru) Special details

**Table:**

Experimental. Data was collected using a Rigaku Synergy S Diffractometer diffractometer equipped with an Oxford 800 low-temperature apparatus. A suitable crystal was chosen and mounted on a nylon loop using Paratone oil. Data were measured based on the Pre-Experiment plugin of CrysAlisPro software. Cell parameters were retrieved using CrysAlisPro software and data reduction was performed using the intergration software inside the CrysAlisPro which corrects for Lp. The structure was solved by the direct method using the SHELXT program and refined by least squares method on F2, SHELXL, incorporated in OLEX2.
Geometry. All esds (except the esd in the dihedral angle between two l.s. planes) are estimated using the full covariance matrix. The cell esds are taken into account individually in the estimation of esds in distances, angles and torsion angles; correlations between esds in cell parameters are only used when they are defined by crystal symmetry. An approximate (isotropic) treatment of cell esds is used for estimating esds involving l.s. planes.
Refinement. The structure was refined by Least Squares SHELXL incorporated in Olex2 software program. All non-hydrogen atoms were refined anisotropically. Hydrogen atom positions were calculated geometrically and refined using the riding model, except for the hydrogen atom on the non-carbon atom(s) which were found by difference Fourier methods and refined isotropically when data permits.

### Bis(µ-trifluoroacetato-κ2O:O')bis{dicarbonyl[tris(pentafluorophenyl)phosphine-κP}diruthenium(I)(Ru—Ru) Fractional atomic coordinates and isotropic or equivalent isotropic displacement parameters (Å^2^)

**Table:**

	*x*	*y*	*z*	*U*_iso_*/*U*_eq_	
Ru1	0.56165 (2)	0.31709 (2)	0.66963 (2)	0.01044 (4)	
Ru2	0.46516 (2)	0.30348 (2)	0.59995 (2)	0.01118 (4)	
P1	0.65530 (2)	0.29811 (2)	0.72510 (2)	0.01115 (8)	
P2	0.39021 (2)	0.26497 (2)	0.53420 (2)	0.01127 (8)	
F1	0.60712 (9)	0.11553 (8)	0.54899 (7)	0.0473 (4)	
F2	0.64080 (7)	0.19946 (8)	0.49985 (5)	0.0323 (3)	
F3	0.69667 (7)	0.17516 (10)	0.56431 (6)	0.0453 (4)	
F4	0.44123 (6)	0.05657 (7)	0.65103 (5)	0.0286 (3)	
F5	0.54339 (7)	0.05827 (7)	0.67269 (6)	0.0369 (3)	
F6	0.46785 (7)	0.08362 (7)	0.72587 (5)	0.0335 (3)	
F7	0.66835 (6)	0.45858 (6)	0.71755 (4)	0.0207 (2)	
F8	0.68387 (6)	0.56278 (6)	0.78672 (4)	0.0251 (2)	
F9	0.69225 (6)	0.52422 (7)	0.88420 (4)	0.0249 (2)	
F10	0.69007 (6)	0.37980 (6)	0.91021 (4)	0.0199 (2)	
F11	0.67927 (5)	0.27589 (6)	0.84255 (4)	0.0168 (2)	
F12	0.71527 (5)	0.36418 (6)	0.63036 (4)	0.0199 (2)	
F13	0.84202 (6)	0.38180 (7)	0.60724 (4)	0.0247 (2)	
F14	0.93697 (6)	0.31972 (8)	0.66315 (5)	0.0290 (3)	
F15	0.90468 (6)	0.24474 (8)	0.74705 (4)	0.0280 (3)	
F16	0.77832 (5)	0.23423 (7)	0.77469 (4)	0.0205 (2)	
F17	0.71661 (6)	0.14002 (7)	0.70573 (4)	0.0256 (2)	
F18	0.67868 (8)	0.01272 (7)	0.74681 (6)	0.0375 (3)	
F19	0.58145 (8)	0.01047 (7)	0.81596 (6)	0.0409 (4)	
F20	0.51824 (7)	0.13724 (8)	0.84075 (5)	0.0337 (3)	
F21	0.55563 (5)	0.26544 (6)	0.79936 (4)	0.0195 (2)	
F22	0.38199 (6)	0.29023 (6)	0.41455 (4)	0.0192 (2)	
F23	0.32351 (6)	0.39933 (7)	0.36843 (4)	0.0264 (3)	
F24	0.25975 (7)	0.50698 (7)	0.41987 (5)	0.0321 (3)	
F25	0.25870 (6)	0.50310 (7)	0.52038 (5)	0.0277 (3)	
F26	0.32068 (5)	0.39541 (6)	0.56881 (4)	0.0194 (2)	
F27	0.33005 (6)	0.20771 (7)	0.63306 (4)	0.0196 (2)	
F28	0.21797 (6)	0.13560 (6)	0.64729 (4)	0.0207 (2)	
F29	0.13688 (6)	0.10475 (7)	0.57061 (5)	0.0275 (3)	
F30	0.17244 (6)	0.14319 (7)	0.47686 (4)	0.0252 (2)	
F31	0.28720 (6)	0.21052 (7)	0.46076 (4)	0.0207 (2)	
F32	0.49576 (6)	0.30844 (6)	0.46578 (4)	0.0192 (2)	
F33	0.57249 (6)	0.22642 (8)	0.40637 (4)	0.0281 (3)	
F34	0.55299 (7)	0.07829 (9)	0.40048 (5)	0.0375 (3)	
F35	0.45907 (6)	0.01305 (7)	0.45649 (6)	0.0358 (3)	
F36	0.38369 (6)	0.09252 (6)	0.51695 (5)	0.0252 (2)	
O1	0.59976 (7)	0.46092 (8)	0.62300 (5)	0.0241 (3)	
O2	0.48458 (8)	0.40181 (8)	0.74563 (5)	0.0257 (3)	
O3	0.47250 (8)	0.45737 (9)	0.55914 (6)	0.0304 (3)	
O4	0.36260 (7)	0.35779 (8)	0.67127 (5)	0.0217 (3)	
O5	0.61583 (6)	0.24992 (7)	0.61960 (5)	0.0156 (2)	
O6	0.54414 (6)	0.25936 (8)	0.55624 (5)	0.0173 (3)	
O7	0.52562 (6)	0.21136 (7)	0.69310 (5)	0.0150 (2)	
O8	0.46333 (6)	0.19237 (7)	0.62531 (5)	0.0155 (3)	
C1	0.58648 (9)	0.40639 (11)	0.64122 (7)	0.0168 (3)	
C2	0.51286 (9)	0.36975 (10)	0.71617 (7)	0.0171 (3)	
C3	0.47106 (9)	0.39928 (11)	0.57496 (7)	0.0188 (4)	
C4	0.40141 (9)	0.33715 (10)	0.64418 (7)	0.0159 (3)	
C5	0.59476 (8)	0.23654 (10)	0.57725 (6)	0.0143 (3)	
C6	0.63596 (10)	0.18059 (11)	0.54748 (8)	0.0210 (4)	
C7	0.49133 (8)	0.17374 (10)	0.66444 (6)	0.0134 (3)	
C8	0.48526 (10)	0.09156 (11)	0.67861 (7)	0.0205 (4)	
C9	0.66877 (8)	0.36275 (10)	0.77690 (6)	0.0128 (3)	
C10	0.67205 (9)	0.43755 (10)	0.76511 (6)	0.0152 (3)	
C11	0.68092 (9)	0.49222 (10)	0.80002 (7)	0.0174 (3)	
C12	0.68630 (9)	0.47267 (11)	0.84943 (7)	0.0176 (3)	
C13	0.68499 (9)	0.39933 (11)	0.86264 (6)	0.0156 (3)	
C14	0.67761 (8)	0.34560 (10)	0.82678 (6)	0.0132 (3)	
C15	0.74111 (9)	0.29678 (10)	0.70316 (6)	0.0137 (3)	
C16	0.75991 (9)	0.33385 (10)	0.66018 (7)	0.0154 (3)	
C17	0.82542 (9)	0.34316 (11)	0.64735 (7)	0.0183 (4)	
C18	0.87426 (10)	0.31240 (12)	0.67642 (7)	0.0206 (4)	
C19	0.85749 (9)	0.27444 (12)	0.71886 (7)	0.0200 (4)	
C20	0.79222 (9)	0.26843 (11)	0.73217 (6)	0.0168 (3)	
C21	0.64047 (9)	0.20853 (10)	0.75408 (7)	0.0150 (3)	
C22	0.66905 (10)	0.14221 (11)	0.73999 (7)	0.0201 (4)	
C23	0.64946 (11)	0.07552 (11)	0.76043 (8)	0.0256 (4)	
C24	0.59960 (12)	0.07412 (12)	0.79511 (8)	0.0274 (5)	
C25	0.56821 (10)	0.13854 (12)	0.80843 (7)	0.0236 (4)	
C26	0.58818 (9)	0.20374 (10)	0.78726 (7)	0.0174 (4)	
C27	0.35617 (8)	0.33865 (10)	0.49457 (6)	0.0134 (3)	
C28	0.35519 (9)	0.34289 (10)	0.44282 (7)	0.0160 (3)	
C29	0.32451 (9)	0.39951 (11)	0.41770 (7)	0.0194 (4)	
C30	0.29204 (9)	0.45405 (11)	0.44357 (8)	0.0211 (4)	
C31	0.29144 (9)	0.45185 (10)	0.49472 (8)	0.0197 (4)	
C32	0.32338 (9)	0.39542 (10)	0.51914 (7)	0.0153 (3)	
C33	0.31296 (8)	0.21485 (10)	0.54622 (6)	0.0131 (3)	
C34	0.29295 (9)	0.19386 (10)	0.59338 (7)	0.0139 (3)	
C35	0.23432 (9)	0.15708 (10)	0.60170 (7)	0.0156 (3)	
C36	0.19300 (9)	0.14070 (10)	0.56266 (7)	0.0186 (4)	
C37	0.21130 (9)	0.16012 (10)	0.51525 (7)	0.0175 (3)	
C38	0.27035 (9)	0.19580 (10)	0.50758 (7)	0.0152 (3)	
C39	0.43524 (9)	0.20442 (10)	0.49198 (6)	0.0144 (3)	
C40	0.48558 (9)	0.23559 (10)	0.46396 (6)	0.0153 (3)	
C41	0.52516 (10)	0.19415 (12)	0.43316 (7)	0.0202 (4)	
C42	0.51587 (10)	0.11896 (12)	0.43067 (8)	0.0241 (4)	
C43	0.46811 (9)	0.08582 (12)	0.45922 (8)	0.0231 (4)	
C44	0.42883 (9)	0.12825 (11)	0.48981 (7)	0.0175 (3)	

### Bis(µ-trifluoroacetato-κ2O:O')bis{dicarbonyl[tris(pentafluorophenyl)phosphine-κP}diruthenium(I)(Ru—Ru) Atomic displacement parameters (Å^2^)

**Table:**

	*U*^11^	*U*^22^	*U*^33^	*U*^12^	*U*^13^	*U*^23^
Ru1	0.00946 (7)	0.01220 (7)	0.00967 (7)	−0.00077 (4)	−0.00152 (4)	0.00027 (4)
Ru2	0.00904 (7)	0.01452 (7)	0.00999 (7)	0.00052 (4)	−0.00137 (4)	−0.00047 (4)
P1	0.01032 (19)	0.01317 (19)	0.00995 (18)	−0.00010 (14)	−0.00092 (15)	0.00043 (14)
P2	0.01027 (18)	0.01408 (19)	0.00946 (18)	0.00033 (15)	−0.00047 (14)	0.00012 (15)
F1	0.0561 (10)	0.0210 (7)	0.0647 (10)	−0.0064 (6)	0.0270 (8)	−0.0118 (6)
F2	0.0316 (7)	0.0431 (8)	0.0223 (6)	0.0027 (6)	0.0092 (5)	−0.0090 (5)
F3	0.0225 (7)	0.0671 (11)	0.0462 (8)	0.0241 (7)	−0.0091 (6)	−0.0267 (8)
F4	0.0335 (7)	0.0206 (6)	0.0319 (7)	−0.0120 (5)	−0.0109 (5)	−0.0004 (5)
F5	0.0300 (7)	0.0197 (6)	0.0610 (9)	0.0068 (5)	−0.0072 (6)	0.0008 (6)
F6	0.0499 (8)	0.0275 (7)	0.0230 (6)	−0.0143 (6)	−0.0051 (6)	0.0089 (5)
F7	0.0304 (6)	0.0182 (5)	0.0135 (5)	−0.0063 (4)	−0.0038 (4)	0.0039 (4)
F8	0.0370 (7)	0.0135 (5)	0.0247 (6)	−0.0057 (5)	−0.0041 (5)	0.0011 (4)
F9	0.0356 (6)	0.0204 (6)	0.0187 (5)	−0.0060 (5)	−0.0016 (5)	−0.0073 (4)
F10	0.0237 (6)	0.0255 (6)	0.0106 (5)	−0.0029 (5)	−0.0016 (4)	−0.0001 (4)
F11	0.0219 (5)	0.0156 (5)	0.0128 (5)	0.0015 (4)	−0.0023 (4)	0.0020 (4)
F12	0.0143 (5)	0.0285 (6)	0.0169 (5)	0.0018 (4)	−0.0015 (4)	0.0081 (4)
F13	0.0194 (5)	0.0376 (7)	0.0170 (5)	−0.0034 (5)	0.0043 (4)	0.0055 (5)
F14	0.0109 (6)	0.0535 (9)	0.0227 (6)	0.0007 (5)	0.0034 (4)	−0.0010 (5)
F15	0.0149 (5)	0.0458 (7)	0.0233 (6)	0.0080 (5)	−0.0048 (4)	0.0034 (5)
F16	0.0172 (5)	0.0313 (6)	0.0131 (5)	0.0023 (4)	−0.0028 (4)	0.0056 (4)
F17	0.0282 (6)	0.0231 (6)	0.0255 (6)	0.0064 (5)	−0.0004 (5)	−0.0075 (5)
F18	0.0490 (8)	0.0154 (6)	0.0481 (8)	0.0051 (6)	−0.0158 (7)	−0.0044 (5)
F19	0.0570 (9)	0.0206 (6)	0.0451 (8)	−0.0183 (6)	−0.0161 (7)	0.0146 (6)
F20	0.0317 (7)	0.0395 (8)	0.0299 (6)	−0.0160 (6)	0.0025 (5)	0.0121 (6)
F21	0.0155 (5)	0.0235 (6)	0.0195 (5)	0.0002 (4)	0.0028 (4)	0.0032 (4)
F22	0.0222 (5)	0.0241 (5)	0.0113 (5)	0.0009 (4)	−0.0016 (4)	−0.0006 (4)
F23	0.0314 (6)	0.0316 (6)	0.0163 (5)	−0.0051 (5)	−0.0070 (5)	0.0097 (5)
F24	0.0331 (7)	0.0237 (6)	0.0394 (7)	0.0048 (5)	−0.0079 (6)	0.0158 (5)
F25	0.0279 (6)	0.0188 (5)	0.0366 (7)	0.0077 (5)	−0.0012 (5)	−0.0021 (5)
F26	0.0182 (5)	0.0241 (6)	0.0160 (5)	0.0053 (4)	−0.0005 (4)	−0.0033 (4)
F27	0.0195 (5)	0.0285 (6)	0.0109 (5)	−0.0049 (4)	−0.0007 (4)	0.0004 (4)
F28	0.0228 (5)	0.0220 (5)	0.0173 (5)	−0.0015 (4)	0.0083 (4)	0.0031 (4)
F29	0.0164 (5)	0.0326 (7)	0.0336 (6)	−0.0102 (5)	0.0042 (5)	0.0006 (5)
F30	0.0202 (6)	0.0316 (6)	0.0236 (6)	−0.0076 (5)	−0.0080 (5)	−0.0002 (5)
F31	0.0231 (6)	0.0271 (6)	0.0120 (5)	−0.0077 (5)	−0.0023 (4)	0.0016 (4)
F32	0.0185 (5)	0.0210 (5)	0.0181 (5)	−0.0020 (4)	0.0029 (4)	0.0025 (4)
F33	0.0216 (6)	0.0430 (8)	0.0198 (6)	0.0004 (5)	0.0101 (5)	0.0001 (5)
F34	0.0256 (6)	0.0446 (8)	0.0422 (8)	0.0043 (6)	0.0101 (6)	−0.0268 (6)
F35	0.0264 (6)	0.0200 (6)	0.0610 (9)	0.0006 (5)	0.0033 (6)	−0.0177 (6)
F36	0.0190 (5)	0.0166 (5)	0.0399 (7)	−0.0009 (4)	0.0076 (5)	0.0013 (5)
O1	0.0225 (7)	0.0210 (7)	0.0287 (7)	−0.0069 (5)	−0.0075 (6)	0.0110 (6)
O2	0.0306 (8)	0.0286 (7)	0.0180 (6)	0.0112 (6)	0.0028 (6)	−0.0048 (6)
O3	0.0316 (8)	0.0216 (7)	0.0381 (9)	−0.0060 (6)	−0.0101 (7)	0.0123 (6)
O4	0.0196 (7)	0.0262 (7)	0.0192 (6)	0.0036 (6)	0.0034 (5)	−0.0054 (5)
O5	0.0133 (6)	0.0196 (6)	0.0139 (6)	0.0025 (5)	−0.0015 (5)	−0.0031 (5)
O6	0.0115 (6)	0.0274 (7)	0.0130 (6)	0.0024 (5)	−0.0007 (5)	−0.0033 (5)
O7	0.0153 (6)	0.0147 (6)	0.0150 (6)	−0.0037 (5)	−0.0017 (5)	0.0014 (5)
O8	0.0153 (6)	0.0155 (6)	0.0157 (6)	−0.0001 (5)	−0.0037 (5)	−0.0008 (5)
C1	0.0128 (8)	0.0229 (9)	0.0147 (8)	−0.0001 (7)	−0.0037 (6)	−0.0009 (7)
C2	0.0166 (8)	0.0175 (8)	0.0172 (8)	0.0006 (7)	−0.0048 (7)	0.0026 (7)
C3	0.0131 (8)	0.0260 (10)	0.0173 (8)	−0.0015 (7)	−0.0036 (6)	−0.0001 (7)
C4	0.0148 (8)	0.0169 (8)	0.0161 (8)	−0.0010 (7)	−0.0052 (7)	−0.0005 (7)
C5	0.0110 (7)	0.0173 (8)	0.0145 (8)	−0.0011 (6)	0.0033 (6)	−0.0004 (6)
C6	0.0146 (9)	0.0251 (10)	0.0232 (9)	0.0008 (7)	0.0008 (7)	−0.0073 (7)
C7	0.0103 (8)	0.0145 (8)	0.0153 (8)	−0.0008 (6)	0.0006 (6)	0.0005 (6)
C8	0.0211 (9)	0.0166 (9)	0.0239 (9)	−0.0025 (7)	−0.0051 (8)	0.0012 (7)
C9	0.0105 (7)	0.0156 (8)	0.0123 (8)	0.0000 (6)	−0.0002 (6)	−0.0012 (6)
C10	0.0147 (8)	0.0180 (8)	0.0129 (8)	−0.0016 (6)	−0.0011 (6)	0.0027 (6)
C11	0.0182 (8)	0.0140 (8)	0.0201 (9)	−0.0035 (7)	−0.0008 (7)	0.0004 (7)
C12	0.0174 (8)	0.0185 (9)	0.0168 (8)	−0.0037 (7)	0.0003 (7)	−0.0053 (7)
C13	0.0131 (8)	0.0225 (9)	0.0112 (8)	−0.0007 (7)	−0.0005 (6)	0.0000 (7)
C14	0.0104 (7)	0.0151 (8)	0.0142 (8)	0.0010 (6)	0.0006 (6)	0.0018 (6)
C15	0.0110 (8)	0.0184 (8)	0.0116 (8)	0.0005 (6)	−0.0011 (6)	−0.0021 (6)
C16	0.0133 (8)	0.0204 (8)	0.0125 (7)	0.0019 (7)	−0.0023 (6)	−0.0009 (7)
C17	0.0172 (9)	0.0258 (9)	0.0121 (8)	−0.0011 (7)	0.0021 (7)	−0.0005 (7)
C18	0.0115 (8)	0.0331 (11)	0.0173 (9)	0.0006 (7)	0.0025 (7)	−0.0057 (7)
C19	0.0137 (8)	0.0303 (10)	0.0161 (8)	0.0047 (7)	−0.0051 (7)	−0.0018 (7)
C20	0.0166 (8)	0.0221 (9)	0.0117 (8)	0.0021 (7)	−0.0013 (6)	−0.0001 (7)
C21	0.0154 (8)	0.0152 (8)	0.0143 (8)	−0.0015 (6)	−0.0063 (6)	0.0010 (6)
C22	0.0216 (9)	0.0205 (9)	0.0182 (9)	0.0004 (7)	−0.0070 (7)	−0.0024 (7)
C23	0.0333 (11)	0.0136 (8)	0.0300 (10)	0.0013 (8)	−0.0169 (9)	−0.0023 (7)
C24	0.0359 (11)	0.0188 (9)	0.0275 (10)	−0.0111 (8)	−0.0155 (9)	0.0084 (8)
C25	0.0232 (10)	0.0269 (10)	0.0206 (9)	−0.0095 (8)	−0.0060 (7)	0.0062 (8)
C26	0.0179 (9)	0.0197 (9)	0.0147 (8)	−0.0021 (7)	−0.0063 (7)	0.0033 (7)
C27	0.0104 (7)	0.0153 (8)	0.0143 (8)	−0.0017 (6)	−0.0023 (6)	0.0016 (6)
C28	0.0141 (8)	0.0170 (8)	0.0170 (8)	−0.0030 (7)	−0.0006 (6)	0.0018 (7)
C29	0.0187 (9)	0.0215 (9)	0.0179 (9)	−0.0078 (7)	−0.0042 (7)	0.0077 (7)
C30	0.0184 (9)	0.0174 (9)	0.0274 (10)	−0.0017 (7)	−0.0078 (7)	0.0096 (7)
C31	0.0167 (9)	0.0144 (8)	0.0280 (10)	0.0003 (7)	−0.0024 (7)	0.0003 (7)
C32	0.0133 (8)	0.0170 (8)	0.0158 (8)	−0.0015 (6)	−0.0015 (6)	0.0001 (7)
C33	0.0108 (7)	0.0148 (8)	0.0137 (8)	0.0002 (6)	0.0001 (6)	0.0000 (6)
C34	0.0144 (8)	0.0141 (8)	0.0132 (8)	0.0014 (6)	−0.0004 (6)	−0.0006 (6)
C35	0.0169 (8)	0.0144 (8)	0.0156 (8)	0.0027 (7)	0.0054 (6)	0.0010 (6)
C36	0.0127 (8)	0.0167 (8)	0.0264 (10)	−0.0019 (7)	0.0028 (7)	−0.0003 (7)
C37	0.0143 (8)	0.0181 (9)	0.0200 (9)	−0.0016 (7)	−0.0038 (7)	−0.0016 (7)
C38	0.0158 (8)	0.0158 (8)	0.0140 (8)	0.0018 (7)	0.0009 (7)	0.0010 (6)
C39	0.0118 (8)	0.0193 (9)	0.0122 (8)	0.0018 (6)	−0.0019 (6)	−0.0016 (6)
C40	0.0152 (8)	0.0196 (9)	0.0110 (7)	−0.0002 (7)	−0.0021 (6)	−0.0015 (6)
C41	0.0157 (9)	0.0324 (11)	0.0125 (8)	0.0001 (7)	0.0004 (7)	−0.0019 (7)
C42	0.0176 (9)	0.0321 (11)	0.0225 (9)	0.0051 (8)	0.0005 (7)	−0.0141 (8)
C43	0.0168 (9)	0.0205 (9)	0.0319 (11)	0.0019 (7)	−0.0036 (8)	−0.0095 (8)
C44	0.0144 (8)	0.0181 (9)	0.0199 (9)	0.0005 (7)	−0.0015 (7)	−0.0016 (7)

### Bis(µ-trifluoroacetato-κ2O:O')bis{dicarbonyl[tris(pentafluorophenyl)phosphine-κP}diruthenium(I)(Ru—Ru) Geometric parameters (Å, º)

**Table:**

Ru1—Ru2	2.7275 (2)	F34—C42	1.333 (2)
Ru1—P1	2.4449 (4)	F35—C43	1.335 (2)
Ru1—O5	2.1262 (12)	F36—C44	1.340 (2)
Ru1—O7	2.1482 (13)	O1—C1	1.137 (2)
Ru1—C1	1.8617 (19)	O2—C2	1.141 (2)
Ru1—C2	1.8636 (19)	O3—C3	1.137 (3)
Ru2—P2	2.4407 (4)	O4—C4	1.139 (2)
Ru2—O6	2.1460 (13)	O5—C5	1.245 (2)
Ru2—O8	2.1289 (13)	O6—C5	1.245 (2)
Ru2—C3	1.868 (2)	O7—C7	1.245 (2)
Ru2—C4	1.8640 (19)	O8—C7	1.247 (2)
P1—C9	1.8457 (18)	C5—C6	1.541 (3)
P1—C15	1.8403 (18)	C7—C8	1.544 (3)
P1—C21	1.8285 (18)	C9—C10	1.395 (2)
P2—C27	1.8464 (18)	C9—C14	1.395 (2)
P2—C33	1.8417 (18)	C10—C11	1.380 (3)
P2—C39	1.8280 (18)	C11—C12	1.385 (3)
F1—C6	1.318 (3)	C12—C13	1.378 (3)
F2—C6	1.335 (2)	C13—C14	1.382 (3)
F3—C6	1.317 (2)	C15—C16	1.395 (3)
F4—C8	1.325 (2)	C15—C20	1.398 (2)
F5—C8	1.335 (2)	C16—C17	1.385 (3)
F6—C8	1.333 (2)	C17—C18	1.382 (3)
F7—C10	1.342 (2)	C18—C19	1.380 (3)
F8—C11	1.331 (2)	C19—C20	1.377 (3)
F9—C12	1.331 (2)	C21—C22	1.389 (3)
F10—C13	1.337 (2)	C21—C26	1.392 (3)
F11—C14	1.335 (2)	C22—C23	1.388 (3)
F12—C16	1.331 (2)	C23—C24	1.379 (3)
F13—C17	1.334 (2)	C24—C25	1.379 (3)
F14—C18	1.329 (2)	C25—C26	1.375 (3)
F15—C19	1.337 (2)	C27—C28	1.400 (2)
F16—C20	1.335 (2)	C27—C32	1.394 (3)
F17—C22	1.338 (2)	C28—C29	1.380 (3)
F18—C23	1.336 (2)	C29—C30	1.379 (3)
F19—C24	1.336 (2)	C30—C31	1.382 (3)
F20—C25	1.339 (3)	C31—C32	1.380 (3)
F21—C26	1.340 (2)	C33—C34	1.390 (2)
F22—C28	1.338 (2)	C33—C38	1.399 (2)
F23—C29	1.331 (2)	C34—C35	1.383 (3)
F24—C30	1.327 (2)	C35—C36	1.380 (3)
F25—C31	1.336 (2)	C36—C37	1.379 (3)
F26—C32	1.343 (2)	C37—C38	1.378 (3)
F27—C34	1.334 (2)	C39—C40	1.392 (3)
F28—C35	1.334 (2)	C39—C44	1.389 (3)
F29—C36	1.330 (2)	C40—C41	1.379 (3)
F30—C37	1.339 (2)	C41—C42	1.379 (3)
F31—C38	1.337 (2)	C42—C43	1.377 (3)
F32—C40	1.338 (2)	C43—C44	1.382 (3)
F33—C41	1.338 (2)		
			
P1—Ru1—Ru2	165.582 (12)	C20—C15—P1	121.79 (14)
O5—Ru1—Ru2	83.17 (3)	F12—C16—C15	121.10 (16)
O5—Ru1—P1	84.64 (3)	F12—C16—C17	116.86 (16)
O5—Ru1—O7	81.49 (5)	C17—C16—C15	122.01 (17)
O7—Ru1—Ru2	83.01 (3)	F13—C17—C16	120.68 (17)
O7—Ru1—P1	87.60 (4)	F13—C17—C18	119.50 (17)
C1—Ru1—Ru2	89.36 (5)	C18—C17—C16	119.82 (17)
C1—Ru1—P1	99.47 (5)	F14—C18—C17	119.57 (18)
C1—Ru1—O5	95.54 (7)	F14—C18—C19	120.67 (18)
C1—Ru1—O7	172.08 (6)	C19—C18—C17	119.77 (18)
C1—Ru1—C2	88.62 (8)	F15—C19—C18	119.84 (17)
C2—Ru1—Ru2	97.48 (5)	F15—C19—C20	120.57 (17)
C2—Ru1—P1	94.13 (6)	C20—C19—C18	119.58 (17)
C2—Ru1—O5	175.79 (7)	F16—C20—C15	119.74 (16)
C2—Ru1—O7	94.45 (7)	F16—C20—C19	117.68 (16)
P2—Ru2—Ru1	167.331 (12)	C19—C20—C15	122.58 (17)
O6—Ru2—Ru1	82.92 (3)	C22—C21—P1	125.72 (15)
O6—Ru2—P2	87.63 (4)	C22—C21—C26	116.17 (17)
O8—Ru2—Ru1	82.89 (3)	C26—C21—P1	117.16 (14)
O8—Ru2—P2	87.27 (4)	F17—C22—C21	121.11 (18)
O8—Ru2—O6	80.62 (5)	F17—C22—C23	117.12 (18)
C3—Ru2—Ru1	96.84 (6)	C23—C22—C21	121.76 (19)
C3—Ru2—P2	92.48 (6)	F18—C23—C22	120.4 (2)
C3—Ru2—O6	95.75 (7)	F18—C23—C24	119.81 (19)
C3—Ru2—O8	176.37 (7)	C24—C23—C22	119.76 (19)
C4—Ru2—Ru1	91.53 (5)	F19—C24—C23	120.3 (2)
C4—Ru2—P2	97.32 (5)	F19—C24—C25	119.7 (2)
C4—Ru2—O6	173.44 (6)	C25—C24—C23	120.05 (19)
C4—Ru2—O8	95.27 (7)	F20—C25—C24	120.36 (19)
C4—Ru2—C3	88.36 (8)	F20—C25—C26	120.6 (2)
C9—P1—Ru1	119.36 (6)	C26—C25—C24	119.0 (2)
C15—P1—Ru1	122.72 (6)	F21—C26—C21	118.74 (16)
C15—P1—C9	96.45 (8)	F21—C26—C25	118.15 (18)
C21—P1—Ru1	105.04 (6)	C25—C26—C21	123.11 (19)
C21—P1—C9	105.33 (8)	C28—C27—P2	128.60 (14)
C21—P1—C15	106.37 (8)	C32—C27—P2	115.93 (13)
C27—P2—Ru2	116.62 (6)	C32—C27—C28	115.34 (16)
C33—P2—Ru2	122.94 (6)	F22—C28—C27	121.64 (16)
C33—P2—C27	98.10 (8)	F22—C28—C29	115.73 (16)
C39—P2—Ru2	108.31 (6)	C29—C28—C27	122.58 (18)
C39—P2—C27	105.09 (8)	F23—C29—C28	119.79 (18)
C39—P2—C33	103.84 (8)	F23—C29—C30	120.08 (17)
C5—O5—Ru1	121.19 (11)	C30—C29—C28	120.05 (18)
C5—O6—Ru2	119.35 (11)	F24—C30—C29	120.72 (18)
C7—O7—Ru1	119.76 (11)	F24—C30—C31	119.90 (19)
C7—O8—Ru2	121.42 (12)	C29—C30—C31	119.34 (17)
O1—C1—Ru1	177.74 (16)	F25—C31—C30	120.18 (17)
O2—C2—Ru1	177.91 (17)	F25—C31—C32	120.11 (18)
O3—C3—Ru2	177.63 (17)	C32—C31—C30	119.69 (18)
O4—C4—Ru2	179.8 (2)	F26—C32—C27	119.67 (16)
O5—C5—C6	114.91 (16)	F26—C32—C31	117.31 (16)
O6—C5—O5	129.61 (17)	C31—C32—C27	123.00 (17)
O6—C5—C6	115.42 (16)	C34—C33—P2	123.07 (13)
F1—C6—F2	106.98 (17)	C34—C33—C38	115.81 (16)
F1—C6—C5	109.41 (16)	C38—C33—P2	121.12 (13)
F2—C6—C5	111.95 (17)	F27—C34—C33	121.32 (16)
F3—C6—F1	109.76 (19)	F27—C34—C35	116.51 (16)
F3—C6—F2	106.44 (17)	C35—C34—C33	122.17 (17)
F3—C6—C5	112.13 (16)	F28—C35—C34	120.35 (17)
O7—C7—O8	129.30 (17)	F28—C35—C36	119.44 (17)
O7—C7—C8	114.83 (15)	C36—C35—C34	120.20 (17)
O8—C7—C8	115.81 (16)	F29—C36—C35	120.19 (17)
F4—C8—F5	108.19 (17)	F29—C36—C37	120.39 (17)
F4—C8—F6	107.92 (16)	C37—C36—C35	119.39 (17)
F4—C8—C7	112.14 (16)	F30—C37—C36	120.12 (17)
F5—C8—C7	109.66 (16)	F30—C37—C38	120.27 (17)
F6—C8—F5	107.47 (17)	C38—C37—C36	119.60 (17)
F6—C8—C7	111.28 (16)	F31—C38—C33	119.89 (16)
C10—C9—P1	116.86 (13)	F31—C38—C37	117.29 (16)
C14—C9—P1	127.57 (14)	C37—C38—C33	122.80 (17)
C14—C9—C10	115.55 (16)	C40—C39—P2	117.53 (14)
F7—C10—C9	119.49 (16)	C44—C39—P2	125.25 (14)
F7—C10—C11	117.19 (16)	C44—C39—C40	116.74 (17)
C11—C10—C9	123.29 (17)	F32—C40—C39	119.65 (16)
F8—C11—C10	120.82 (17)	F32—C40—C41	118.04 (17)
F8—C11—C12	120.18 (17)	C41—C40—C39	122.30 (18)
C10—C11—C12	118.99 (17)	F33—C41—C40	120.42 (18)
F9—C12—C11	120.49 (17)	F33—C41—C42	120.30 (18)
F9—C12—C13	119.82 (17)	C42—C41—C40	119.27 (19)
C13—C12—C11	119.69 (17)	F34—C42—C41	120.0 (2)
F10—C13—C12	120.23 (16)	F34—C42—C43	119.9 (2)
F10—C13—C14	119.67 (17)	C43—C42—C41	120.05 (18)
C12—C13—C14	120.10 (16)	F35—C43—C42	119.84 (18)
F11—C14—C9	121.53 (16)	F35—C43—C44	120.28 (19)
F11—C14—C13	116.21 (15)	C42—C43—C44	119.87 (19)
C13—C14—C9	122.25 (17)	F36—C44—C39	121.45 (17)
C16—C15—P1	121.38 (13)	F36—C44—C43	116.87 (17)
C16—C15—C20	116.15 (16)	C43—C44—C39	121.67 (18)
			
Ru1—P1—C9—C10	−53.63 (15)	O7—C7—C8—F6	50.3 (2)
Ru1—P1—C9—C14	127.94 (14)	O8—C7—C8—F4	−11.1 (2)
Ru1—P1—C15—C16	25.80 (18)	O8—C7—C8—F5	109.11 (19)
Ru1—P1—C15—C20	−164.02 (13)	O8—C7—C8—F6	−132.12 (17)
Ru1—P1—C21—C22	99.35 (16)	C9—P1—C15—C16	−105.42 (16)
Ru1—P1—C21—C26	−68.99 (14)	C9—P1—C15—C20	64.75 (16)
Ru1—O5—C5—O6	3.0 (3)	C9—P1—C21—C22	−133.77 (16)
Ru1—O5—C5—C6	−173.87 (12)	C9—P1—C21—C26	57.88 (15)
Ru1—O7—C7—O8	−14.1 (3)	C9—C10—C11—F8	179.83 (17)
Ru1—O7—C7—C8	163.10 (12)	C9—C10—C11—C12	−0.6 (3)
Ru2—P2—C27—C28	129.05 (15)	C10—C9—C14—F11	−176.22 (15)
Ru2—P2—C27—C32	−55.44 (15)	C10—C9—C14—C13	3.9 (3)
Ru2—P2—C33—C34	−1.75 (18)	C10—C11—C12—F9	−177.32 (17)
Ru2—P2—C33—C38	177.97 (12)	C10—C11—C12—C13	2.2 (3)
Ru2—P2—C39—C40	−67.41 (14)	C11—C12—C13—F10	−179.93 (16)
Ru2—P2—C39—C44	104.37 (16)	C11—C12—C13—C14	−0.7 (3)
Ru2—O6—C5—O5	−19.3 (3)	C12—C13—C14—F11	177.64 (16)
Ru2—O6—C5—C6	157.54 (12)	C12—C13—C14—C9	−2.5 (3)
Ru2—O8—C7—O7	−3.5 (3)	C14—C9—C10—F7	175.71 (15)
Ru2—O8—C7—C8	179.31 (12)	C14—C9—C10—C11	−2.4 (3)
P1—C9—C10—F7	−2.9 (2)	C15—P1—C9—C10	79.82 (15)
P1—C9—C10—C11	179.01 (15)	C15—P1—C9—C14	−98.61 (17)
P1—C9—C14—F11	2.2 (3)	C15—P1—C21—C22	−32.13 (18)
P1—C9—C14—C13	−177.63 (14)	C15—P1—C21—C26	159.53 (14)
P1—C15—C16—F12	−8.0 (2)	C15—C16—C17—F13	−176.87 (17)
P1—C15—C16—C17	169.67 (15)	C15—C16—C17—C18	2.9 (3)
P1—C15—C20—F16	7.2 (2)	C16—C15—C20—F16	177.90 (16)
P1—C15—C20—C19	−172.48 (15)	C16—C15—C20—C19	−1.8 (3)
P1—C21—C22—F17	6.9 (3)	C16—C17—C18—F14	178.07 (18)
P1—C21—C22—C23	−172.66 (15)	C16—C17—C18—C19	−1.9 (3)
P1—C21—C26—F21	−5.7 (2)	C17—C18—C19—F15	−179.91 (18)
P1—C21—C26—C25	174.38 (15)	C17—C18—C19—C20	−0.9 (3)
P2—C27—C28—F22	−1.2 (3)	C18—C19—C20—F16	−176.94 (17)
P2—C27—C28—C29	176.09 (14)	C18—C19—C20—C15	2.8 (3)
P2—C27—C32—F26	2.8 (2)	C20—C15—C16—F12	−178.75 (16)
P2—C27—C32—C31	−175.54 (14)	C20—C15—C16—C17	−1.0 (3)
P2—C33—C34—F27	−1.6 (2)	C21—P1—C9—C10	−171.20 (14)
P2—C33—C34—C35	179.14 (14)	C21—P1—C9—C14	10.36 (18)
P2—C33—C38—F31	3.6 (2)	C21—P1—C15—C16	146.49 (15)
P2—C33—C38—C37	−178.17 (14)	C21—P1—C15—C20	−43.34 (17)
P2—C39—C40—F32	−5.2 (2)	C21—C22—C23—F18	−178.78 (17)
P2—C39—C40—C41	176.12 (14)	C21—C22—C23—C24	1.1 (3)
P2—C39—C44—F36	5.4 (3)	C22—C21—C26—F21	−175.20 (15)
P2—C39—C44—C43	−175.33 (15)	C22—C21—C26—C25	4.9 (3)
F7—C10—C11—F8	1.7 (3)	C22—C23—C24—F19	−178.31 (18)
F7—C10—C11—C12	−178.74 (16)	C22—C23—C24—C25	1.7 (3)
F8—C11—C12—F9	2.2 (3)	C23—C24—C25—F20	177.65 (18)
F8—C11—C12—C13	−178.22 (17)	C23—C24—C25—C26	−1.1 (3)
F9—C12—C13—F10	−0.4 (3)	C24—C25—C26—F21	177.75 (17)
F9—C12—C13—C14	178.82 (16)	C24—C25—C26—C21	−2.4 (3)
F10—C13—C14—F11	−3.2 (2)	C26—C21—C22—F17	175.38 (16)
F10—C13—C14—C9	176.72 (16)	C26—C21—C22—C23	−4.2 (3)
F12—C16—C17—F13	0.9 (3)	C27—P2—C33—C34	−130.90 (16)
F12—C16—C17—C18	−179.30 (17)	C27—P2—C33—C38	48.82 (16)
F13—C17—C18—F14	−2.2 (3)	C27—P2—C39—C40	57.90 (15)
F13—C17—C18—C19	177.86 (18)	C27—P2—C39—C44	−130.31 (16)
F14—C18—C19—F15	0.1 (3)	C27—C28—C29—F23	−177.95 (16)
F14—C18—C19—C20	179.17 (18)	C27—C28—C29—C30	−1.3 (3)
F15—C19—C20—F16	2.1 (3)	C28—C27—C32—F26	178.87 (15)
F15—C19—C20—C15	−178.16 (17)	C28—C27—C32—C31	0.6 (3)
F17—C22—C23—F18	1.6 (3)	C28—C29—C30—F24	−176.88 (17)
F17—C22—C23—C24	−178.53 (17)	C28—C29—C30—C31	0.9 (3)
F18—C23—C24—F19	1.5 (3)	C29—C30—C31—F25	−178.15 (17)
F18—C23—C24—C25	−178.45 (18)	C29—C30—C31—C32	0.2 (3)
F19—C24—C25—F20	−2.4 (3)	C30—C31—C32—F26	−179.28 (16)
F19—C24—C25—C26	178.93 (18)	C30—C31—C32—C27	−0.9 (3)
F20—C25—C26—F21	−1.0 (3)	C32—C27—C28—F22	−176.75 (15)
F20—C25—C26—C21	178.92 (17)	C32—C27—C28—C29	0.6 (3)
F22—C28—C29—F23	−0.5 (2)	C33—P2—C27—C28	−97.66 (17)
F22—C28—C29—C30	176.14 (16)	C33—P2—C27—C32	77.84 (14)
F23—C29—C30—F24	−0.3 (3)	C33—P2—C39—C40	160.42 (14)
F23—C29—C30—C31	177.54 (17)	C33—P2—C39—C44	−27.80 (18)
F24—C30—C31—F25	−0.3 (3)	C33—C34—C35—F28	177.64 (16)
F24—C30—C31—C32	177.99 (17)	C33—C34—C35—C36	−0.8 (3)
F25—C31—C32—F26	−1.0 (3)	C34—C33—C38—F31	−176.66 (16)
F25—C31—C32—C27	177.38 (17)	C34—C33—C38—C37	1.6 (3)
F27—C34—C35—F28	−1.7 (3)	C34—C35—C36—F29	179.41 (17)
F27—C34—C35—C36	179.90 (16)	C34—C35—C36—C37	1.2 (3)
F28—C35—C36—F29	1.0 (3)	C35—C36—C37—F30	178.53 (17)
F28—C35—C36—C37	−177.21 (17)	C35—C36—C37—C38	−0.3 (3)
F29—C36—C37—F30	0.3 (3)	C36—C37—C38—F31	177.10 (17)
F29—C36—C37—C38	−178.47 (17)	C36—C37—C38—C33	−1.2 (3)
F30—C37—C38—F31	−1.7 (3)	C38—C33—C34—F27	178.72 (16)
F30—C37—C38—C33	−179.96 (17)	C38—C33—C34—C35	−0.6 (3)
F32—C40—C41—F33	0.5 (3)	C39—P2—C27—C28	9.11 (18)
F32—C40—C41—C42	179.82 (17)	C39—P2—C27—C32	−175.39 (13)
F33—C41—C42—F34	−1.8 (3)	C39—P2—C33—C34	121.29 (16)
F33—C41—C42—C43	178.45 (18)	C39—P2—C33—C38	−58.99 (16)
F34—C42—C43—F35	−0.1 (3)	C39—C40—C41—F33	179.16 (16)
F34—C42—C43—C44	−178.79 (18)	C39—C40—C41—C42	−1.5 (3)
F35—C43—C44—F36	1.9 (3)	C40—C39—C44—F36	177.24 (16)
F35—C43—C44—C39	−177.38 (18)	C40—C39—C44—C43	−3.5 (3)
O5—C5—C6—F1	100.7 (2)	C40—C41—C42—F34	178.90 (18)
O5—C5—C6—F2	−140.89 (17)	C40—C41—C42—C43	−0.9 (3)
O5—C5—C6—F3	−21.3 (2)	C41—C42—C43—F35	179.65 (19)
O6—C5—C6—F1	−76.6 (2)	C41—C42—C43—C44	1.0 (3)
O6—C5—C6—F2	41.8 (2)	C42—C43—C44—F36	−179.42 (18)
O6—C5—C6—F3	161.38 (18)	C42—C43—C44—C39	1.3 (3)
O7—C7—C8—F4	171.27 (16)	C44—C39—C40—F32	−177.74 (16)
O7—C7—C8—F5	−68.5 (2)	C44—C39—C40—C41	3.6 (3)
